# Healthcare Resource Utilization During a Multimodal Nutritional Program with Oral Nutritional Supplements in Malnourished Outpatients †

**DOI:** 10.3390/nu17172854

**Published:** 2025-09-03

**Authors:** Isabel Cornejo-Pareja, Kirk W. Kerr, Maria Ramirez, Maria Camprubi-Robles, Isabel Maria Vegas-Aguilar, Rocio Fernandez-Jimenez, Mar Amaya-Campos, Angela Martinez-Martinez, Jesus Martinez-Martin, Jose Manuel Garcia-Almeida

**Affiliations:** 1 Department of Endocrinology and Nutrition, Virgen de la Victoria Hospital, 29010 Malaga, Spain; isabel.mva13@gmail.com (I.M.V.-A.); mariadelmarac2@gmail.com (M.A.-C.); angela.mar.guadix@gmail.com (A.M.-M.); jgarciaalmeida@gmail.com (J.M.G.-A.); 2 Instituto de Investigación Biomédica de Málaga (IBIMA)-Plataforma BIONAND, 29590 Malaga, Spain; 3 Faculty of Medicine, University of Malaga, 29071 Malaga, Spain; 4 Abbott Nutrition, Research and Development, Columbus, OH 43219, USA; 5 Abbott Nutrition, Research and Development, 18004 Granada, Spain; maria.ramirez@abbott.com (M.R.); maria.camprubirobles@abbott.com (M.C.-R.); 6 Department of Financial Control, Virgen de la Victoria Hospital, 29010 Malaga, Spain; jesus.martinez.sspa@juntadeandalucia.es

**Keywords:** beta-hydroxy-beta-methylbutyrate, health economics, healthcare cost, healthcare resource utilization, oral nutritional supplements, malnutrition, cancer

## Abstract

**Background/Objective:** We compared healthcare utilization and cost outcomes for patients before and after they underwent a nutrition care intervention that included daily consumption of specialized or standard oral nutritional supplements (ONS) as part of a multimodal program. We sought to determine whether this nutritional intervention was associated with reduced use of healthcare resources and lowered costs. **Methods:** This retrospective analysis included adult patients who were referred to the medical nutrition office with malnutrition or its risk. We followed outcomes for patients who had received nutritional interventions (3–6 months) that included diet and exercise recommendations as well as a specialized ONS enriched with β-hydroxy-β-methylbutyrate (HMB-ONS) or a standard ONS (S-ONS). We reviewed hospital records for resource utilization data—hospital (re)admissions, number of days in the hospital, patient visits to the emergency department (ED), and visits to general practitioners (GP) and specialty physician clinics. We compared healthcare utilization for intervals up to 12 months before and after the initiation of the nutrition care intervention. We also examined healthcare utilization and costs as well as changes in oncology treatment following initiations of the nutrition care intervention for a subgroup of cancer patients. **Results:** Over a 12-month period following the start of nutritional intervention with ONS, the patients had lower healthcare resource use and costs. With daily use of S-ONS or HMB-ONS for up to 6 months, the patients’ healthcare utilization was significantly reduced at 3-, 6-, and 12-month time points for hospital admissions, ED visits, GP visits, and specialty visits (all *p* < 0.001). Overall, ONS use was associated with reduced patient healthcare costs by half (EUR 27,024.80 to EUR 13,349.60, *p* < 0.001). Significant clinical predictors of higher use of healthcare resources and costs were older age, having positive indications of malnutrition, exhibiting a greater Charlson Comorbidity Index (CCI), and having been hospitalized for oncology surgery. Regression analysis revealed that ONS intervention was associated with lower total healthcare costs (β_Post-ONS_ = −0.504), and that patients receiving HMB-ONS (β_ONS-HMB_ = −0.583) had a greater reduction in costs than did patients receiving S-ONS in the 12 months after nutritional intervention. Patients with cancer who received HMB-ONS were less likely than those receiving S-ONS to need suspension or dose reduction in oncology drug treatment due to failure or intolerance of the treatment drug. **Conclusions:** multimodal programs including ONS improve health economic outcomes for patients with poor nutritional status due to disease. Health economic outcome improvements included lower healthcare resource use, lower healthcare costs, and, for patients being treated for cancer, a reduced likelihood of treatment failure. The use of ONS enriched with HMB provided advantages over standard ONS, both in health outcomes and cost-of-care reductions.

## 1. Introduction

The relationship between poor nutritional status and poor health is complex and bidirectional. People with poor nutritional status are at increased risk of health complications such as infections, declining physical abilities, and reduced quality of life or even death [1]. Those who experience severe illnesses or injuries are vulnerable to malnutrition due to decreased appetite and lower food intake and to increased macro- and micronutrient needs for recovery and healing processes [2]. Older people also experience aging-related loss of muscle, which can be worsened when illness, injury, and malnutrition are also present [3]. Surgical patients with malnutrition suffer higher postoperative morbidity, experience longer lengths of hospital stay, and have elevated readmission rates [4,5,6,7,8]. If nutrient needs are unmet, physical function can decline, recovery can be delayed, and overall healthcare needs increased with elevated costs for care [2,3,9].

Research has shown that malnutrition remains a significant healthcare challenge in Spain. The PREDyCES study estimated that 23.7% of hospitalized patients were malnourished and that malnutrition was associated with significantly longer hospital stays [10]. The SeDREno study in Spain built on the work of PREDyCES using the Global Leadership In Malnutrition (GLIM) criteria to identify hospitalized patients with malnutrition [11]. The study found that 29.7% of hospitalized patients were malnourished, with even greater prevalence in patients aged 70 years and older. In addition to measuring the prevalence of malnutrition, the PREDyCES and SeDREno studies both identified cancer as an independent risk factor for malnutrition. The prevalence of malnutrition adversely impacted healthcare systems by driving up cost. Research by Rodriguez-Sanchez et al. estimated that patients having malnutrition were associated with 11–13% higher hospitalization costs [12].

Fortunately, substantial evidence now shows improved recovery from illness, injury, surgery, and aging-related physical decline when malnutrition is actively addressed and treated. Daily consumption of macro- and micronutrient-enriched oral nutritional supplements (ONS) has proven highly effective in treating patients with malnutrition in community, nursing home, and hospital settings [13,14,15,16,17,18,19]. Growing evidence shows that improved clinical outcomes are also reflected in cost benefits resulting from reduced use of healthcare resources [8,13,14,20,21].

Adequate supplies of protein, essential amino acids (leucine, arginine, cysteine, and glutamine), vitamin D, and other micronutrients are important to muscle recovery, along with physical activity or exercise [22]. Beta-hydroxy-beta-methylbutyrate (HMB), a leucine metabolite that promotes protein anabolism and inhibits protein catabolism, has gained increasing attention for its role in ameliorating skeletal muscle wasting and weakness [23,24,25]. This metabolic benefit is consistent with reported low endogenous HMB levels in patients with sarcopenia (low muscle mass and function) [26]. When sarcopenic older adults used ONS that contained HMB and vitamin D, better outcomes appeared to reflect improvements in skeletal muscle health [27,28]. Intervention with nutritionally complete ONS with HMB is recognized to lessen risk for adverse consequences of malnutrition and to improve physical function. However, there remains a knowledge gap regarding whether such improved health and function can also yield cost-savings due to reduced utilization of healthcare services.

Our study’s primary objective was to examine the impact of multimodal medical nutrition therapy including diet and exercise recommendations and supplemental nutrition including ONS on healthcare resource use and costs. Our secondary analysis compared healthcare utilization and cost outcomes for people receiving daily specialized ONS with HMB (HMB-ONS) with those for people receiving standard ONS without HMB (S-ONS), to detect potential differences in outcomes based on type of ONS supplementation. Additionally, we examined the impact of ONS on healthcare resource use and costs for a subgroup of patients with oncology diagnoses. Finally, we examined the impact of HMB-ONS, compared to S-ONS, on patients with oncology diagnoses.

## 2. Materials and Methods

### 2.1. Study Design

This retrospective study builds upon the prior work of Cornejo et al. (2021) by analyzing health outcomes and healthcare resource use of patients (Virgen de la Victoria Hospital, Malaga, Spain) whose health records documented referral to the medical nutrition office [29]. These patients were seen in a clinical setting, according to clinical practice of the department, and then by the medical nutrition office to treat a diagnosis of malnutrition and provide needed nutritional treatment. An important proportion of the patients were oncology patients (N = 179). This article is a revised and expanded version of papers entitled “Healthcare Resource Utilization by Malnourished Outpatients declines following Intervention with Oral Nutrition Supplement with β-Hydroxy-β-Methylbutyrate and Vitamin D” and “Intervention with Oral Nutrition Supplements with β-Hydroxy-β-Methylbutyrate and Vitamin D in Malnourished Oncology Outpatients associated with Reduced Healthcare Resource Utilization and Costs”, which was presented at 45th ESPEN Congress on Clinical Nutrition and Metabolism, Lyon, France, 11–14 September 2023 [30,31].

### 2.2. Medical Nutrition Therapy and Multimodal Program

Details on the nutrition intervention, and adherence to it, have been reported previously [29]. Briefly, patients received nutritional counseling with a trained dietitian. Also, patients were advised to consume 2 servings/day of ONS (HMB-ONS or S-ONS) for 3 to 6 months (until the time of next visit) and received strength exercise recommendations using input from the hospital’s Rehabilitation Unit.

### 2.3. Outcome Measures: Healthcare Resources and Costs

For this analysis, cumulative healthcare resource use and costs were compared for periods of 3, 6, and 12 months before and after initial nutritional therapy. The data on healthcare resource use was collected from each patients’ hospital record via chart review. Measures of healthcare resource use included hospital (re)admissions, patient visits to the emergency department, visits to a general practitioner, and visits to a specialist physician. Healthcare resource use was totaled for periods of 3, 6, and 12 months before and after the initial nutritional therapy.

Healthcare costs were estimated using the average cost of each type of visit (Supplementary Table S1) and the number of visits made by a patient. Individualized calculations of costs generated by hospital admission episodes were computed using the Diagnosis-Related Groups (DRG) tool. This tool allows for the correlation of different types of patients treated in a hospital with the cost of their care. The tool determines the level of complexity of each episode considering the main diagnosis, the service or specialty where the admission takes place, the use of support services, and the number of days of admission. Subsequently, the hospital’s cost service provided the specific cost of each episode, adjusted according to the costs established in the hospital and in accordance with the national healthcare system [32]. Other cost outcomes were estimated by using the average cost of each type of visit according to the Spanish Ministry of Health and the number of visits made by a patient [32]. Total healthcare costs were calculated as the sum of costs from each care setting.

We estimated the cost of the ONS interventions and compared these to the estimated savings from reduced healthcare resource use. We estimated the cost of S-ONS and HMB-ONS using price per serving of market leading products, EUR 3.36 and EUR 4.65, respectively. Assuming patients used ONS twice a day for up to 6 months, the estimated costs of S-ONS and HMB-ONS are EUR 1209.6 and EUR 1672.6, respectively, and the incremental cost of HMB-ONS versus S-ONS is EUR 463. The benefits of HMB-ONS are estimated from the cost savings from reduced healthcare resource use by multiplying the marginal effect of HMB-ONS on each healthcare resource use by the cost of that resource. To simplify estimating cost savings from specialist visits, we utilized the median cost of specialist visits (EUR 310) across all specialist types in place of estimating costs for each specialty.

### 2.4. Impact on Oncology Treatment

Since a high proportion of study patients had a diagnosis of cancer (63%) (See Supplementary Table S2 for cancer types), we also analyzed changes in oncology treatment to determine their relationship with nutrition care and healthcare outcomes. A physician classified treatment regimen changes as reduction in dose or suspension of treatment. Suspension of treatment or reduction in dose were notable because these adjustments indicated that the patient’s condition had deteriorated or that the patient had poor tolerance of the treatment drug. The changes were identified by their timing in relation to the initial nutritional therapy (i.e., at 3, 6 or 12 months before or after initial nutritional therapy). Changes in treatment were analyzed jointly as whether a patient had either a reduction in dose or a suspension of treatment compared with no change in oncology therapy to allow for sufficient variation in the outcome variable.

### 2.5. Statistical Analyses

Baseline demographics were reported as mean values with standard deviation (SD) for continuous variables (age, body mass index (BMI), Charlson Comorbidity Index (CCI)) and as number (N) and percent total (%) for categorical variables (sex, reason for nutrition therapy). Healthcare resource use and costs over 3-, 6-, and 12-month periods after the beginning of nutritional therapy were compared to use in the corresponding interval prior to initial nutritional therapy. Simple group comparisons were conducted using Student’s *t*-test [33].

The relationship between outcome variables (healthcare resource use, healthcare costs, oncology treatment changes) and explanatory variables of patients’ sex (β_male_), age (β_age_), BMI (β_BMI_), malnutrition diagnosis (β_maln_), CCI (β_CCI_), and initial admission for oncology or general surgery (β_ONC_, β_GenSurg_) were examined using regression analysis. Βeta represents the change in the outcome variable for a one-unit change in the explanatory variable; β was determined by regression analysis to control for differential impact these factors may have had on patient outcomes. The natural logarithm of healthcare costs was used as the dependent variable in cost regressions to correct for data skewness.

In addition to patient-specific explanatory variables, fixed effects were included for the period after initial nutrition therapy (β_Post-ONS_) and for patients who were provided standard ONS or ONS with HMB (β_ONS-Type_). The post-ONS fixed effect controls for differences in outcomes between the periods before and after initial nutrition therapy that are not accounted for by other independent variables. The ONS-type fixed effect controls for differences in outcomes due to differences in patients receiving standard ONS versus ONS with HMB that are not accounted for by other independent variables. The difference-in-differences approach employs an interaction of the post-ONS treatment variable (β_Post-ONS_) and ONS-type variable (β_ONS-Type_) to estimate the impact of HMB-ONS on outcomes (β_ONS-HMB_) independent of the impact of standard ONS [34,35]. Poisson regression was used to analyze the number of hospital admissions, emergency department visits, primary care visits, and specialist visits; ordinary least squares regression was used for number of hospital days. Marginal effects (mfx) of HMB-ONS on outcomes of Poisson regressions were calculated using the average value of the dependent variable. For oncology patients, changes in treatment (reduction in dose or suspension of treatment) were analyzed using logistic regression. The parameters were considered statistically significant at the 5% level (i.e., *p*-value ≤ 0.05).

## 3. Results

### 3.1. Healthcare Utilization Analysis

Average healthcare resource use and healthcare costs 3, 6, and 12 months before and after intervention with ONS medical nutrition therapy are compared in Table 1. The average number of hospital admissions per patient declined from 1.53 in the 12 months prior to receiving the ONS intervention to 0.5 in the 12 months following the intervention (a 67.3% reduction). Likewise, ED visits were reduced by 48%, from 3.48 to 1.81; GP visits were reduced by 37.1% from 6.12 to 3.85; and specialist visits were reduced by 16.6%, from 10.32 to 8.61 in the period after nutrition intervention. Although we have presented the 12-month results in the text, reductions in all types of healthcare resource use were also observed at 3 and 6 months after nutrition intervention.

Average costs per patient were also lower after ONS intervention (Table 1). Average total costs per patient declined from EUR 27 024.8 in the 12 months prior to ONS intervention to EUR 13 349.6 in the 12 months after intervention (50.6%). ED costs were reduced from EUR 805.1 to EUR 410.2, showing a 49% reduction; GP costs declined by 37.1% from EUR 265.2 to EUR 166.8; and specialist costs declined by 17.6% from EUR 8336.7 to EUR 6872.6 in the period after ONS intervention. Significant cost reductions were also observed 3 and 6 months after nutrition intervention.

Comparability of the demographic characteristics HMB-ONS and S-ONS groups was examined in previous research [29]. We do note that CCI, a measure of severity of illness with higher numbers indicating greater severity, was slightly higher in the S-ONS group, but the difference was not statistically significant (Supplementary Table S3).

### 3.2. Regression Analysis of Healthcare Resource Use

Regression analysis was employed to examine the impact of multiple factors on healthcare resource use, particularly the impact of HMB-ONS. Outcome variables of hospital readmissions, ED visits, GP visits, and medical specialist visits over 12 months before and after the ONS initiation were regressed on the independent variables (Table 2). Patients with a diagnosis of malnutrition had more hospital readmissions (β_maln_ = 0.29) and GP visits (β_maln_ = 0.09) that not malnourished patients. Patients receiving ONS with HMB had 0.73 fewer hospital readmissions (β_ONS-HMB_ = −0.71, mfx = −0.73), 1.3 fewer ED visits (β_ONS-HMB_ = −0.49, mfx = −1.31), and 1.64 fewer GP visits (β_ONS-HMB_ = −0.33, mfx = −1.64) controlling for other factors. Higher (more severe) CCI was associated greater healthcare resource use across hospital readmissions (β_CCI_ = 0.05), ED visits (β_CCI_ = 0.07), and GP (β_CCI_ = 0.03) and specialist visits (β_CCI_ = 0.02). Patients admitted for oncology or general surgery had fewer ED visits (β_onc-surg_ = −0.29; β_gen-surg_ = −0.29), and more GP visits (β_gen-surg_ = 0.19).

### 3.3. Cost Analysis

**Figure 1:**
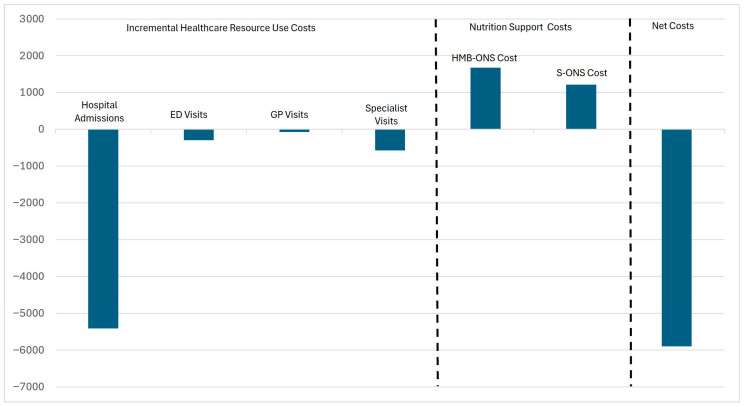
Cost analysis of healthcare resource use and nutrition support for HMB-ONS versus S-ONS. Note: Negative values indicate savings.

Using the estimates from the regressions on the impact of HMB-ONS, we estimate the cost savings from using HMB-ONS compared to S-ONS. Estimated incremental costs savings per patient using HMB-ONS are EUR 5412.6 from reduced hospitalizations, EUR 299.9 for reduced emergency room visits, EUR 71.3 from reduced primary care visits, and EUR 575.5 from reduced specialist visits, for a total cost savings of EUR 6359. Deducting the incremental cost of HMB-ONS over S-ONS from the estimated cost savings from reduced healthcare utilization results in an estimated net cost savings of EUR 5896 per patient (Figure 1).

### 3.4. Healthcare Resource Utilization of Oncology Patients

Oncology patients are a population of special interest due to the vulnerability of their health, nutrition, and healthcare resource needs. Regression analysis was employed to examine the impact of several factors on healthcare resource use of oncology patients (Table 3). Patients with diagnosed malnutrition had more GP visits over 12 months (β_maln_ = 0.13). Oncology patients receiving ONS with HMB had 1.14 fewer hospital readmissions (β_ONS-HMB_ = −1.08, mfx = −1.14), 1.98 fewer ED visits (β_ONS-HMB_ = −0.768, mfx = −1.98), 2.29 fewer GP visits (β_ONS-HMB_ = −0.447, mfx = −2.29) and 2.21 fewer specialist visits (β_ONS-HMB_ = −0.20; mfx = −2.21). Patients with higher (more severe) CCI had greater healthcare resource use across hospital readmissions (β_CCI_ = 0.05), ED visits (β_CCI_ = 0.08), and GP (β_CCI_ = 0.03) and specialist visits (β_CCI_ = 0.02).

### 3.5. Cost Comparison for Oncology Patients

**Figure 2:**
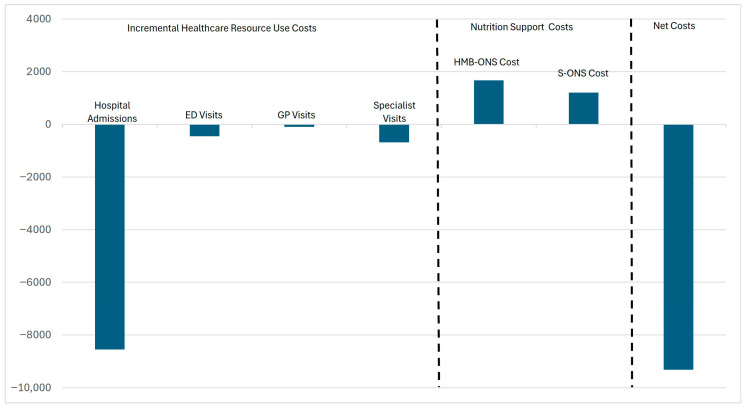
Cost analysis of healthcare resource use and nutrition support for HMB-ONS versus S-ONS in oncology patients. Note: Negative values indicate savings.

Using the same methodology employed for the full patient population, we estimated the savings from reduced healthcare resource use for oncology patients using HMB-ONS compared to patients S-ONS and compared these to the incremental cost of HMB-ONS over S-ONS. Cost savings per patient from reduced healthcare resource use are EUR 8542.5 from reduced hospitalizations, EUR 455.5 from reduced emergency room visits, EUR 99.6 from reduced primary care visits, and EUR 684.8 from reduced specialist visits for total cost savings of EUR 9782.4. Net cost savings, defined as total cost savings less the EUR 463 incremental cost of HMB-ONS, are estimated to be EUR 9319.4 per patient (Figure 2).

### 3.6. Regression Analysis of Oncology Treatment Changes

Next, we analyzed the impact of the nutrition intervention on oncology treatment changes over 12 months following the start of nutrition intervention (Final column of Table 3). Patients who received HMB-ONS intervention had eight times lower odds of reduction in dose or suspension of oncological treatment versus S-ONS over 12 months (β_ONS-HMB_ = −2.144; OR = 0.117). This result was not significant at *p* < 0.05 after Bonferroni adjustment, but it demonstrates a trend of reduction in oncology treatment changes in patients receiving HMB-ONS. Patients with a higher CCI score (indicating more severe morbidity) were more likely to have either a reduction in treatment-drug dose or a suspension of treatment (β_CCI_ = 0.192).

## 4. Discussion

Healthcare systems are faced with high and increasing costs for care. With rising costs and declining affordability, public and private healthcare payers are looking for value in care. Our main study findings showed evidence of outcome benefits for ONS use, in particular HMB-ONS, as part of interventional nutrition care. Our initial study showed benefits of ONS in terms of improved nutritional status [29]. Our present analysis extended these findings to show the value of nutritional interventions with standard-ONS and HMB-ONS in terms of lowered need for using healthcare resources, which translated to lower costs of care. We have also demonstrated that patients who received nutritional intervention including HMB-ONS had even lower healthcare resource use over the 12-month follow-up period than did patients receiving an S-ONS. Better outcomes at lower costs represent value in healthcare. Notably, the benefits of ONS use in our study extended beyond the treatment time, as we continued to measure health benefits up to 12 months, even when nutritional treatment was only 3 to 6 months. It is also important to note that while some factors related to healthcare resource use, such as patient age and comorbidities, cannot be immediately influenced by practitioners, provision of ONS is a factor that healthcare provides can influence and implement with relative ease.

### 4.1. Nutrition Interventions’ Impact on Healthcare Resource Use

Taken together, our findings are consistent with previous studies of nutrition interventions’ impact on healthcare resource use across a variety of care settings. Schuetz et al. conducted a randomized, controlled trial of nutrition care for malnourished hospitalized patients; results showed reduced ICU length of stay, fewer complications, and cost savings of EUR 199 per hospitalization [19]. A meta-analysis of randomized, controlled trials of nutritional support during hospitalization and after discharge found cost savings of USD 2818 per patient [20]. Sriram et al. and Sulo et al. found that a hospital-based multimodal nutrition-focused quality improvement program (QIP), which focused on timely identification of patients at risk for malnutrition, and rapid intervention with dietitian consultation and disease-specific ONS resulted in significant reductions in patient length of hospital stay, 30-day readmission rates, and costs savings of USD 3858 per patient over 30 days [36,37]. A follow-up nutrition-focused QIP conducted in a home-healthcare setting found that a nutrition intervention including ONS resulted in reduced hospitalization and cost-savings of USD 1558 per patient over 30 days [14,38]. Hong et al. found that a nutrition-focused QIP for outpatients reduced hospitalizations, GP visits, and specialist visits, resulting in cost savings of USD 485 over 90 days [13]. Other studies have found that ONS reduced hospitalization, and improved quality of life [8,39]. In contrast to the relatively short periods studied previously, our study estimated cost savings of over EUR 5000 per patient from reductions in healthcare resource use up to 1 year after initial nutrition intervention, approximately equivalent to savings of EUR 1250 in 90 days, and 420 euros in 30 days. Consistent with the literature, our study demonstrates that provision of ONS to malnourished patients improves patient outcomes and reduces healthcare costs.

### 4.2. The Impact of ONS with HMB

A unique aspect of our studies was their examination of the role of HMB-ONS in improving health and nutrition status and its impact on healthcare costs. Prior studies have focused on ONS more generally, rather than a particular type of ONS. We estimated that the use of HMB-ONS reduced healthcare spending by more than its cost, resulting in net cost savings. Previous health economic analyses of HMB-ONS illustrated the cost-effectiveness of HMB-ONS intervention. Zhong et al.’s analysis of the NOURISH trial found that providing HMB-ONS to malnourished hospitalized patients reduced patient mortality and estimated the cost-effectiveness ratio of HMB-ONS intervention to be USD 33,818 per quality-adjusted life year, well within the typical bounds of cost-effectiveness [21]. Ballesteros-Pomar et al. applied the results of the same clinical trial to the Spanish National Health System and found that HMB-ONS was cost-effective, with a cost per life-year gained over 90 days of EUR 34,700 [39]. Thus, our study contributes to the growing body of literature on HMB-ONS as a cost-effective intervention by arguing that HMB-ONS may be cost saving, a higher standard than cost-effectiveness. This study is also unique in comparing outcomes of patients receiving HMB-ONS with outcomes of patients using S-ONS, and finding HMB-ONS to be cost saving compared to S-ONS. Our study also presents evidence that patients receiving HMB-ONS were less likely to have their oncology treatment suspended or dose reduced. This finding builds on prior research showing that ONS combined with nutritional counseling resulted in reduced need for dose reduction or treatment suspension in head and neck cancer patients [40]. Improving patient tolerance to anticancer therapies is of great importance as the side effects and toxicity of oncology treatments can prevent patients from receiving treatment on their physician designed schedule [41]. Our present results, combined with the results of our previous study showing improvements in oncology nutrition status [29], suggest that further research on nutrition in supporting oncology patients is needed, particularly on patients’ tolerance of and availability for treatment.

### 4.3. Strengths and Limitations

Key strengths of this study are its detailed, individualized data on patient health status and healthcare resource use. Health status, in particular nutrition status, was assessed by qualified personnel with treatment follow-up. Additionally, the 12-month time frame of the study enables us to observe the effect of nutritional therapy on healthcare resource use and costs over time. Furthermore, our analysis of oncology patients’ treatments is unique in examining the impact of nutritional intervention on treatment progression. Future research is needed to examine the connection between nutrition interventions, health economic outcomes, and oncology treatment when patients are randomly or quasi-randomly assigned to different nutritional interventions.

Nonetheless, this study has limitations common to other retrospective studies. The data describing patients’ medical conditions were detailed but may be limited in ways that might confound our findings due to unobserved factors. The type of ONS was not randomly assigned to patients, thus it is not possible to solely attribute the reduction in healthcare resource use and costs observed in patients receiving HMB-ONS to the type of ONS they received. The study was limited to patients treated at a single center, potentially limiting the applicability of the findings to other populations. Additionally, the costs utilized in the cost analysis are drawn from Spain’s national health system and may differ from costs in other jurisdictions.

## 5. Conclusions

In summary, the results of our study showed that patients had lower healthcare resource use and healthcare costs over a 12-month interval after a multimodal intervention—which included diet and exercise recommendations and daily ONS for up to 6 months—compared with resource use prior to the nutritional intervention. Furthermore, patients who received nutritional intervention including HMB-ONS had even lower healthcare resource use and costs over the 12-month follow-up period than did patients receiving an S-ONS. Patients with cancer who were receiving HMB-ONS had an even lower likelihood that their oncology treatment would need to be suspended, or dosage reduced, than did patients receiving S-ONS. Our analysis provides evidence that intervention with ONS, especially HMB-ONS, offers benefits for health outcomes and for cost-savings in people who were found to have poor nutritional status. Future research should explore the impact of HMB-ONS on cancer-related outcomes and patient quality of life, particularly in their tolerance of, and availability for, treatment. Additionally, future randomized controlled trials of HMB-ONS should monitor health economic variables such as healthcare resource use and quality of life. Finally, quality improvement projects are needed to demonstrate how to implement a scalable program using HMB-ONS in inpatient and outpatient settings.

## Figures and Tables

**Table 1:** Healthcare resource use and costs of all patients 3, 6, and 12 months before and after any ONS intervention (N_patients_ = 283).

Time	0–3 Months			0–6 Months			0–12 Months		
Cost per Patient	Before	After	%Δ	Before	After	%Δ	Before	After	%Δ
# of Hospital admissions	0.64 (0.71)	0.17 (0.44)	73.4 *	1.07 (0.90)	0.31 (0.64)	71.0 *	1.53 (1.09)	0.50 (0.87)	67.3 *
# of ED visits	1.18 (1.79)	0.58 (1.15)	50.8 *	2.16 (2.57)	1.03 (1.77)	52.3 *	3.48 (3.39)	1.81 (2.43)	48.0 *
# of GP Visits	1.62 (1.80)	1.14 (1.39)	29.6 *	3.33 (3.14)	2.17 (2.34)	34.8 *	6.12 (4.61)	3.85 (3.46)	37.1 *
# of Specialist visits	4.30 (2.82)	2.76 (2.40)	35.8 *	7.04 (4.75)	5.41 (4.11)	23.2 *	10.32 (7.12)	8.61 (6.13)	16.6 *
Total costs	14,471.2 (17,488.4)	5749.2 (12,475.9)	60.3 *	20,679.1 (19,207.8)	9129.5 (14,163.3)	55.9 *	27,024.8 (21,505.1)	13,349.6 (16,651.7)	50.6 *
ED costs	275.4 (413.2)	131.6 (253.6)	52.2 *	495.5 (589.7)	234.0 (401.7)	52.8 *	805.1 (782.5)	410.2 (555.7)	49.0 *
GP costs	69.94 (79.06)	49.5 (60.62)	29.2 *	143.9 (136.2)	93.5 (101.8)	35.0 *	265.2 (200.5)	166.8 (151.0)	37.1 *
Specialist costs	3314.6 (3646.9)	2260.3 (2718.1)	31.8 *	5738.0 (6242.8)	4363.0 (5102.5)	24.0 *	8336.7 (8727.5)	6872.6 (7612.2)	17.6 *

Data are mean (SD) before and after the intervention. Costs are in euros. *: significantly different from ‘before intervention’ at *p* < 0.01.

**Table 2:** Regressions of healthcare resource use over 12 months before and after ONS initiation. Healthcare resource utilization (hospital admissions, emergency department visits, GP visits, medical specialist visits, and total days in the hospital) over 12 months before and after the nutritional therapy initiation regressed on the dependent variables using Poisson regression. N_patients_ = 281.

	Dependent Variables			
	Hospital Admissions 12 Months	Emergency Dept Visits 12 Months	GP Visits 12 Months	Medical Specialist Visits 12 Months
Independent Variables	Estimate (Std Error)	Estimate (Std Error)	Estimate (Std Error)	Estimate (Std Error)
Intercept	0.13 (0.31)	1.90 * (0.19)	1.61 * (1.14)	2.58 * (0.11)
Male	0.11 (0.09)	−0.05 (0.05)	−0.15 * (0.04)	0.07 * (0.03)
Age	−0.01 * (0.004)	−0.01 * (0.002)	−0.003 (0.002)	−0.005 * (0.001)
BMI	0.004 (0.01)	−0.001 (0.01)	−0.001 (0.005)	−0.02 * (0.004)
Malnourished	0.29 * (0.10)	0.11 (0.06)	0.09 * (0.04)	−0.07 (0.03)
CCI	0.05 * (0.02)	0.07 * (0.01)	0.03 * (0.01)	0.02 * (0.01)
Admitted for Oncology surgery	0.02 (0.12)	−0.29 * (0.07)	0.07 (0.06)	0.47 * (0.04)
Admitted for General Surgery	0.07 (0.15)	−0.29 * (0.09)	0.19 * (0.07)	−0.08 (0.06)
Post-ONS	−0.53 (0.23)	−0.25 (0.13)	−0.19 (0.10)	−0.003 (0.07)
ONS Type	0.31 (0.15)	0.03 (0.09)	0.22 * (0.07)	0.12 (0.06)
ONS-HMB	−0.71 * (0.26)	−0.49 * (0.14)	−0.33 * (0.11)	−0.20 (0.08)

*—Significant at *p* ≤ 0.05 after Bonferroni adjustment.

**Table 3:** Regressions of oncology patient healthcare resource use over 12 months before and after ONS initiation. Healthcare resource utilization (hospital admissions, emergency ED visits, GP visits, medical specialist visits, and total days in the hospital) of oncology patients over 12 months before and after the nutritional therapy initiation regressed on independent variables using Poisson regression. Logistic regression of oncology treatment change (reduced dose or suspension of treatment) regressed on independent variables. N_patients_ = 177.

	Dependent Variables				
	Hospital Admissions 12 Months	Emergency Dept Visits 12 Months	GP Visits 12 Months	Medical Specialist Visits 12 Months	Reduce Dose or Suspend Treatment 12 Months
Independent Variables	Estimate (Std Error)	Estimate (Std Error)	Estimate (Std Error)	Estimate (Std Error)	Estimate (Std Error)
Intercept	−0.56 (0.46)	1.36 * (0.28)	1.67 * (0.20)	3.06 * (0.14)	−2.30 (1.29)
Male	0.31 * (0.11)	0.01 (0.07)	−0.20 * (0.05)	0.13 * (0.03)	−0.21 (0.28)
Age	−0.009 (0.006)	−0.02 * (0.004)	−0.01 (0.003)	−0.008 * (0.002)	−0.01 (0.02)
BMI	0.02 (0.01)	0.01 (0.01)	0.01 (0.01)	−0.01 * (0.004)	0.01 (0.04)
Malnourished	0.10 (0.13)	−0.02 (0.08)	0.13 (0.06)	−0.03 (0.04)	0.39 (0.35)
CCI	0.05 (0.02)	0.08 * (0.01)	0.03 * (0.01)	0.02 * (0.01)	0.19 * (0.06)
Post-ONS	−0.15 (0.31)	0 (0.16)	−0.04 (0.14)	−0.05 (0.09)	1.31 (0.81)
ONS Type	0.63 * (0.22)	0.06 (0.12)	0.30 * (0.10)	0.14 * (0.07)	0.53 (0.72)
ONS-HMB	−1.08 * (0.34)	−0.77 * (0.18)	−0.45 * (0.15)	−0.20 * (0.10)	−2.14 (0.86)

*—Significant at *p* ≤ 0.05 with Bonferroni Adjustment.

## Data Availability

Data are contained within the article and Supplementary Materials.

## Supplementary Materials

The following supporting information can be downloaded at https://www.mdpi.com/article/10.3390/nu17172854/s1: Table S1: Mean cost per patient per unit visit or service; Table S2: Types of cancer diagnosed in patients; Table S3: Patient demographic and descriptive characteristics by treatment group; Table S4: Regressions of healthcare resource use over 6 months before and after ONS initiation; Table S5: Regressions of healthcare resource use over 3 months before and after ONS initiation; Table S6: Regressions of oncology patient healthcare resource use over 6 months before and after ONS initiation; Table S7: Regressions of oncology patient healthcare resource use over 3 months before and after ONS initiation.
