# Supplementary material for: Healthcare Resource Utilization During a Multimodal Nutritional Program with Oral Nutritional Supplements in Malnourished Outpatients
Source: Nutrients. 2025 Sep 3;17(17):2854. doi: 10.3390/nu17172854 (PMC12429871; doi:10.3390/nu17172854)
Supplement: Supplementary file 1 [file nutrients-17-02854-s001.zip › nutrients-3820107-supplementary.pdf]

Supplementary Materials

Supplementary Table S1. Mean cost per patient per unit visit or service

Type of visit or service	Mean unit costs (Euros €)
Hospital Stay	7462*
Hospital Emergency Room Visit	229.9
Primary Care Physician Visit	43.5
Specialist Physician Visit	
Anesthesia	121
Vascular Medicine	164
Cardiac Surgery	505
General Surgery	194
Urology	180
Gynecology	107
Trauma	105
Ophthalmology	106
Ear, Nose, Throat	231
Cardiology	296
Gastroenterology	107
Endocrinology & Nutrition	348
Internal Medicine	274
Infectious Diseases	464
Pulmonology	371
Neurology	194
Nephrology	171
Rheumatology	185
Rehabilitation	288
Allergy	262
Dermatology	71
Hematology	369
Medical Oncology	1532
Radiation Oncology	780
Mental Health	382

*Median value was used instead of mean value due to data skewness
†Mean value across all specialties

Supplementary Table S2: Types of cancer diagnosed in patients

Type of Cancer	Number of patients (% of cancer patients)
Blood & Lymphatic System	1 (0.6%)
Breast	4 (2.2%)
Digestive System	124 (69.3%)
Endocrine System	2 (1.1%)
Head & Neck	27 (15.1%)
Lymphoma	1 (0.6%)
Reproductive System	13 (7.3%)
Respiratory System	3 (1.7%)
Skin	1 (0.6%)
Urinary System	3 (1.7%)

Supplementary Table S3. Patient demographic and descriptive characteristics by treatment group

Abbreviations: HMB: β-hydroxy-β-methylbutyrate; N: number of observations; SD, standard deviation

	Overall, N=283	HMB-ONS, N=240	Standard-ONS, N=43	p-values (between-group)
Age in years as mean (SD)	60.9 (14.2)	59.0 (14.6)	62.7 (13.8)	0.131
Body Mass Index, mean (SD)	23.2 (4.4)	23.2 (4.5)	23.1 (4.3)	0.827
Charlson Comorbidity Index, mean (SD)	4.7 (3.0)	4.6 (2.9)	5.0 (3.1)	0.383
Sex, N (%)				
Male	149 (53%)	121 (50.4%)	28 (65.0%)	0.107
Female	134 (47%)	119 (49.6%)	15 (34.9%)	
Admission reason, N (%)				
Oncology surgery	179 (63%)	155 (64.6%)	24 (55.8%)	0.304
General surgery	44 (16%)	34 (14.2%)	10 (23.3%)	
Other	60 (21%)	51 (21.2%)	9 (20.9%)	

Supplementary Table S4. Regressions of healthcare resource use over 6 months before and after ONS initiation

Health care resource utilization (hospital readmissions, emergency department visits, GP visits, medical specialist visits, and total days in the hospital) over 6 months before and after the initial hospitalization regressed on the dependent variables using Poisson regression. $N_{patients}$ = 281.

	**Dependent variables**			
	**Total Hospital Readmissions during 6-mo**	**Total Emergency Dept Admissions during 6-mo**	**Total GP Visits during 6-mo**	**Total Medical Specialist Visits during 6-mo**
**Independent Variables**	Estimate (Std Error)	Estimate (Std Error)	Estimate (Std Error)	Estimate (Std Error)
Intercept	-0.32 (0.38)	0.74* (0.24)	0.900* (0.19)	2.09* (0.13)
Male	0.05 (0.10)	0.04 (0.07)	-0.11 (0.05)	0.09* (0.03)
Age	-0.01* (0.01)	-0.01* (0.003)	-0.01 (0.003)	-0.005* (0.002)
BMI	0.02 (0.01)	0.02* (0.01)	0.003 (0.01)	-0.02* (0.004)
Malnourished	0.38* (0.13)	0.19 (0.08)	0.17* (0.06)	0.02 (0.04)
CCI	0.05 (0.03)	0.08* (0.01)	0.04* (0.01)	0.03* (0.01)
Admitted for Oncology	-0.02 (0.15)	-0.28* (0.09)	0.09 (0.08)	0.45* (0.05)
Admitted for General Surgery	0.06 (0.17)	-0.32* (0.12)	0.16 (0.09)	-0.09 (0.07)
Post ONS	-0.75* (0.29)	-0.33 (0.17)	-0.16 (0.14)	-0.09 (0.09)
ONS Type	0.23 (0.18)	0.05 (0.12)	0.23 (0.10)	0.03 (0.07)
ONS-HMB	-0.58 (0.32)	-0.49* (0.19)	-0.32 (0.15)	-0.19 (0.10)

*-Significant at p≤0.05 after Bonferroni adjustment

Supplementary Table S5. Regressions of healthcare resource use over 3 months before and after ONS initiation

Health care resource utilization (hospital readmissions, emergency department visits, GP visits, medical specialist visits, and total days in the hospital) over 3 months before and after the initial hospitalization regressed on the dependent variables using Poisson regression. $N_{patients}$ = 281.

	**Dependent variables**			
	**Total Hospital Readmissions during 3-mo**	**Total Emergency Dept Admissions during 3-mo**	**Total GP Visits during 3-mo**	**Total Medical Specialist Visits during 3-mo**
**Independent Variables**	Estimate (Std Error)	Estimate (Std Error)	Estimate (Std Error)	Estimate (Std Error)
Intercept	-1.51* (0.50)	0.08 (0.33)	0.09 (0.27)	1.50* (0.17)
Male	0.10 (0.13)	0.01 (0.09)	-0.23* (0.07)	0.08 (0.05)
Age	-0.01 (0.01)	-0.02* (0.004)	-0.01 (0.004)	-0.004 (0.002)
BMI	0.03 (0.02)	0.03* (0.01)	0.01 (0.01)	-0.01 (0.01)
Malnourished	0.34 (0.16)	0.18 (0.11)	0.20 (0.09)	0.06 (0.05)
CCI	0.04 (0.03)	0.08* (0.02)	0.03 (0.02)	0.04* (0.01)
Admitted for Oncology	0.21 (0.21)	-0.19 (0.13)	0.16 (0.11)	0.27* (0.07)
Admitted for General Surgery	0.30 (0.23)	-0.27 (0.16)	0.20 (0.13)	-0.20 (0.09)
Post ONS	-0.89* (0.40)	-0.48 (0.23)	-0.09 (0.19)	-0.39* (0.12)
ONS Type	0.22 (0.23)	-0.06 (0.16)	0.12 (0.14)	-0.05 (0.08)
ONS-HMB	-0.51 (0.43)	-0.26 (0.25)	-0.31 (0.20)	-0.07 (0.13)

*-Significant at p≤0.05 after Bonferroni adjustment

Supplementary Table S6.

Regressions of oncology patient healthcare resource use over 6 months before and after ONS initiation

Health care resource utilization (hospital admissions, emergency department visits, GP visits, medical specialist visits, and total days in the hospital) of oncology patients over 6 months before and after the nutritional therapy initiation regressed on independent variables using Poisson regression. Oncology treatment change (reduced dose or suspension of treatment) regressed on independent variables. $N_{patient}$ = 177.

	**Dependent Variables**				
	**Total Hospital Admissions 6-mo**	**Total Emergency Dept Visits 6-mo**	**Total GP Visits 6-mo**	**Total Medical Specialist Visits 6-mo**	**Reduce dose or suspend treatment 6-mo**
**Independent variables**	Estimate (Std Error)	Estimate (Std Error)	Estimate (Std Error)	Estimate (Std Error)	Estimate (Std Error)
Intercept	-1.14 (0.55)	0.32 (0.35)	1.15* (0.27)	2.64* (0.16)	-2.91 (1.20)
Male	0.16 (0.13)	0.24* (0.09)	-0.17* (0.06)	0.16* (0.04)	-0.41 (0.28)
Age	-0.01 (0.01)	-0.02* (0.004)	-0.01* (0.003)	-0.01* (0.002)	-0.004 (0.02)
BMI	0.03 (0.02)	0.03* (0.01)	0.004 (0.01)	-0.01 (0.01)	0.04 (0.04)
Malnourished	0.33 (0.17)	0.14 (0.11)	0.20* (0.08)	0.06 (0.05)	0.75 (0.37)
CCI	0.06 (0.03)	0.11* (0.02)	0.05 (0.01)	0.02 (0.01)	0.16* (0.06)
Post-ONS	-0.44 (0.39)	-0.25 (0.21)	-0.11 (0.18)	-0.17 (0.104)	0.39 (0.70)
ONS Type	0.44 (0.26)	-0.03 (0.15)	0.22 (0.13)	-0.04 (0.08)	-0.13 (0.59)
ONS-HMB	-0.69 (0.42)	-0.53 (0.29)	-0.31 (0.19)	-0.15 (0.11)	-1.13 (0.77)

*-Significant at p≤0.05 after Bonferroni adjustment

Supplementary Table S7.

Regressions of oncology patient healthcare resource use over 3 months before and after ONS initiation

Health care resource utilization (hospital admissions, emergency department visits, GP visits, medical specialist visits, and total days in the hospital) of oncology patients over 3 months before and after the nutritional therapy initiation regressed on independent variables using Poisson regression. Oncology treatment change (reduced dose or suspension of treatment) regressed on independent variables. $N_{patient}$ = 175

	**Dependent Variables**				
	**Total Hospital Admissions 3-mo**	**Total Emergency Dept Visits 3-mo**	**Total GP Visits 3-mo**	**Total Medical Specialist Visits 3-mo**	**Reduce dose or suspend treatment 3-mo**
**Independent variables**	Estimate (Std Error)	Estimate (Std Error)	Estimate (Std Error)	Estimate (Std Error)	Estimate (Std Error)
Intercept	-1.99* (0.70)	-0.36 (0.47)	0.55 (0.37)	1.70* (0.22)	-4.12* (1.34)
Male	0.16 (0.17)	0.23 (0.12)	-0.24* (0.09)	0.16* (0.054)	-0.62 (0.32)
Age	-0.01 (0.01)	-0.02* (0.01)	-0.01 (0.01)	-0.003 (0.003)	-0.02 (0.02)
BMI	0.04* (0.02)	0.04* (0.01)	0.02 (0.01)	-0.01 (0.01)	0.11* (0.04)
Malnourished	0.33 (0.21)	0.26 (0.14)	0.27* (0.11)	0.12 (0.07)	0.84 (0.42)
CCI	0.05 (0.04)	0.11* (0.02)	0.04* (0.02)	0.03* (0.01)	0.14 (0.07)
Post-ONS	-0.54 (0.48)	-0.19 (0.28)	-0.17 (0.24)	-0.51* (0.14)	0.97 (0.72)
ONS Type	0.28 (0.31)	-0.03 (0.20)	-0.001 (0.18)	-0.18 (0.09)	0.01 (0.61)
ONS-HMB	-0.61 (0.52)	-0.49 (0.31)	-0.21 (0.26)	0.07 (0.15)	-1.75 (0.80)

*-Significant at p≤0.05 after Bonferroni adjustment
